# Release of Anti-Inflammatory Palmitoleic Acid and Its Positional Isomers by Mouse Peritoneal Macrophages

**DOI:** 10.3390/biomedicines8110480

**Published:** 2020-11-06

**Authors:** Alma M. Astudillo, Clara Meana, Miguel A. Bermúdez, Alfonso Pérez-Encabo, María A. Balboa, Jesús Balsinde

**Affiliations:** 1 Instituto de Biología y Genética Molecular, Consejo Superior de Investigaciones Científicas (CSIC), Universidad de Valladolid, 47003 Valladolid, Spain; alma@ibgm.uva.es (A.M.A.); cmeana@ibgm.uva.es (C.M.); mabermudez@ibgm.uva.es (M.A.B.); mbalboa@ibgm.uva.es (M.A.B.); 2 Centro de Investigación Biomédica en Red de Diabetes y Enfermedades Metabólicas Asociadas (CIBERDEM), 28029 Madrid, Spain; 3 Instituto CINQUIMA, Departamento de Química Orgánica, Universidad de Valladolid, 47011 Valladolid, Spain; alfonso.perez.encabo@uva.es

**Keywords:** palmitoleic acid, phospholipid hydrolysis, phospholipase A_2_, lipid mediators, fatty acid esters of hydroxy fatty acids, inflammation, monocytes/macrophages

## Abstract

Positional isomers of hexadecenoic acid are considered as fatty acids with anti-inflammatory properties. The best known of them, palmitoleic acid (cis-9-hexadecenoic acid, 16:1n-7), has been identified as a lipokine with important beneficial actions in metabolic diseases. Hypogeic acid (cis-7-hexadecenoic acid, 16:1n-9) has been regarded as a possible biomarker of foamy cell formation during atherosclerosis. Notwithstanding the importance of these isomers as possible regulators of inflammatory responses, very little is known about the regulation of their levels and distribution and mobilization among the different lipid pools within the cell. In this work, we describe that the bulk of hexadecenoic fatty acids found in mouse peritoneal macrophages is esterified in a unique phosphatidylcholine species, which contains palmitic acid at the sn-1 position, and hexadecenoic acid at the sn-2 position. This species markedly decreases when the macrophages are activated with inflammatory stimuli, in parallel with net mobilization of free hexadecenoic acid. Using pharmacological inhibitors and specific gene-silencing approaches, we demonstrate that hexadecenoic acids are selectively released by calcium-independent group VIA phospholipase A_2_ under activation conditions. While most of the released hexadecenoic acid accumulates in free fatty acid form, a significant part is also transferred to other phospholipids to form hexadecenoate-containing inositol phospholipids, which are known to possess growth-factor-like-properties, and are also used to form fatty acid esters of hydroxy fatty acids, compounds with known anti-diabetic and anti-inflammatory properties. Collectively, these data unveil new pathways and mechanisms for the utilization of palmitoleic acid and its isomers during inflammatory conditions, and raise the intriguing possibility that part of the anti-inflammatory activity of these fatty acids may be due to conversion to other lipid mediators.

## 1. Introduction

Accumulating evidence suggests a key role for the cis-unsaturated fatty acid palmitoleic acid (cis-9-hexadecenoic acid; 16:1n-7) in protecting against hepatic steatosis and improving overall insulin sensitivity in metabolic tissues [1,2]. In murine models, strong evidence is available to support a role for palmitoleic acid as an anti-inflammatory mediator and a positive correlate of insulin sensitivity. In humans, however, opposing results have been reported. Some studies have indicated that circulating levels of palmitoleate positively correlate with insulin sensitivity and decreased inflammatory markers, while others showed that the circulating levels of palmitoleate correlate with the degree of hepatic steatosis and adiposity, which promotes fat deposition in hepatocytes. Clearly, more research in humans is needed to validate the promising therapeutic potential of palmitoleic acid suggested by murine and in vitro studies [1,2,3,4].

While the direct actions of 16:1n-7 on cells of innate immunity remain largely unexplored, it appears clear that this fatty acid manifests marked anti-inflammatory effects [5,6]. Notably, recent work has demonstrated that innate immune cells also contain significant levels of a positional isomer of palmitoleic acid, cis-7-hexadecenoic acid (16:1n-9; hypogeic acid), which also manifests strong anti-inflammatory properties on monocytes and macrophages [7,8,9]. Furthermore, 16:1n-9 is synthesized by human phagocytic cells via β-oxidation of oleic acid and its levels are elevated in lipid droplet-laden monocytes, suggesting that it may constitute a biomarker for foamy cell formation [8]. When added exogenously, 16:1n-9 exhibits potent anti-inflammatory actions in vitro and in vivo, which are distinguishable from those of 16:1n-7 and comparable in magnitude to those exerted by omega-3 fatty acids [8,9]. Monocytes and macrophages also contain significant quantities of a third 16:1 positional isomer, cis-6-hexadecenoic acid (16:1n-10; sapienic acid). 16:1n-10 also exhibits anti-inflammatory activity, although generally at higher concentrations than those observed for 16:1n-7 or 16:1n-9 [9].

It is well established that fatty acyl chains of membrane lipids play a variety of biological functions including signaling; thus, the enzymes regulating phospholipid fatty acid recycling constitute a key step for the fine regulation of lipid mediator production during cell activation. Phospholipase A_2_ (PLA_2_) enzymes, which cleave the sn-2 position of glycerophospholipids, are key regulators of these processes [10,11,12]. Multiple PLA_2_ enzymes co-exist in a single cell, each exhibiting potentially different headgroup and/or fatty acid preferences. PLA_2_s are classified in 16 groups, each containing several sub-groups [13,14]. However, based on biochemical features, these enzymes are frequently grouped into six major families: secreted PLA_2_s, Ca^2+^-independent PLA_2_s, cytosolic PLA_2_s, platelet activating factor acetyl hydrolases (also known as lipoprotein-associated PLA_2_), lysosomal PLA_2_, and the adipose PLA_2_ [13,14]. Of these families, two of them have been shown to play key roles in regulating fatty acid mobilization reactions in innate immune cells, namely the cytosolic group IVA phospholipase A_2_ (cPLA_2_α) and the Ca^2+^-independent group VIA phospholipase A_2_ (iPLA_2_-VIA). cPLA_2_α is the critical enzyme regulating stimulus-induced arachidonic acid release in cells under a wide variety of conditions [15,16]. iPLA_2_-VIA is a multifaceted enzyme with multiple roles in cell physiology and pathophysiology, being of special relevance in regulating bone formation, apoptosis, insulin secretion, and sperm development [17,18]. Unlike cPLA_2_α, iPLA_2_-VIA does not manifest specificity with regard to the fatty acid released in in vitro assays [10,13]. However, studies carried out in murine macrophages have shown that cPLA_2_α and iPLA_2_-VIA act on different cellular phospholipid pools [19,20]. While the former regulates arachidonic acid release and eicosanoid production, the latter appears to act on phospholipids that do not contain arachidonate [19,20]. In this regard, we show in this paper, for the first time, that macrophages do release 16:1 fatty acids when challenged by activating stimuli, and that the process appears to be exquisitely regulated by iPLA_2_-VIA. Moreover, we also show that part of the 16:1 fatty acids initially present in choline-containing glycerophospholipids (PC) are transferred to phosphatidylinositol (PI) and/or used to produce 16:1-containing branched fatty acid esters of hydroxy fatty acids (FAHFAs), a family of compounds that displays significant anti-inflammatory activity in vivo [21].

## 2. Materials and Methods

### 2.1. Reagents

Cell culture medium was from Corning (Glendale, AZ, USA). Organic solvents were from Fisher Scientific (Madrid, Spain). Lipid standards were from Avanti Polar Lipids (Alabaster, AL, USA) or Larodan Fine Chemicals (Malmö, Sweden). PLA_2_ inhibitors were from Cayman (Ann Arbor, MI, USA). The Coenzyme A-independent transacylase inhibitor SKF98625 [22,23] was synthesized in our laboratory. Deuterated 9-cis-hexadecenoic acid (16:1n-7) was from Cayman, and deuterated cis-7-hexadecenoic acid (16:1n-9) was synthesized in our laboratory. The detailed procedure is described in Appendix A. All other reagents were from Sigma-Aldrich (Madrid, Spain). 

### 2.2. Cell Culture

Resident peritoneal macrophages from Swiss male mice (University of Valladolid Animal House, 10–12 weeks old) were obtained by peritoneal lavage using 5 mL cold phosphate-buffered saline, as described elsewhere [24]. The cells were plated at 2 × 10^6^ per well (6-well plates) in 2 mL Roswell Park Memorial Institute 1640 medium with 10% heat-inactivated fetal bovine serum, 100 U/mL penicillin, and 100 μg/mL streptomycin, and allowed to adhere for 20 h in a humidified atmosphere of 5% CO_2_ at 37 °C. Wells were extensively washed to remove nonadherent cells. Adherent macrophages were then used for experimentation. All procedures involving animals were carried out under the supervision of the Institutional Committee of Animal Care and Usage of the University of Valladolid (approval number 7406000; date: 04/19/2016; renewed: 10/08/2019), and are in accordance with the guidelines established by the Spanish Ministry of Agriculture, Food, and Environment and the European Union. For the antisense inhibition experiments, RAW264.7 macrophage-like cells were used. These cells were grown in Dulbecco’s modified Eagle’s medium supplemented with 10% (*v*/*v*) fetal bovine serum, 100 U/mL penicillin, 100 µg/mL streptomycin, and 2 mM L-glutamine at 37 °C in a humidified atmosphere of 5% CO_2_ at 37 °C, as previously described [25,26]. For experiments, the cells were treated in serum-free medium for 1 h before addition of stimulants for the indicated concentrations and periods of time. When inhibitors were used, they were added to the media 30 min before stimulating the cells. Zymosan was prepared as described [27]. Only zymosan preparations that showed no endogenous PLA_2_ activity, as measured by enzyme assay [28,29,30,31], were used in this study. Protein was measured using a commercial kit (BioRad, Hercules, CA, USA). 

### 2.3. Gas Chromatography/Mass Spectrometry (GC/MS) Analyses

Total lipids from approximately 10^7^ cells were extracted according to Bligh and Dyer [32], and internal standards were added. Phospholipids were separated from neutral lipids by thin-layer chromatography, using *n*-hexane/diethyl ether/acetic acid (70:30:1, *v*/*v*/*v*) as the mobile phase [33]. Phospholipid classes were separated twice with chloroform/methanol/28% (*w*/*w*) ammonium hydroxide (60:37.5:4, *v*/*v*/*v*) as the mobile phase, using plates impregnated with boric acid [34]. The bands corresponding to the different lipid classes were scraped from the plate, and fatty acid methyl esters were obtained from the various lipid fractions by transmethylation with 0.5 M KOH in methanol for 60 min at 37 °C. Fatty acid methyl esters were analyzed by GC/MS as previously described [35,36], using an Agilent 7890A gas chromatograph coupled to an Agilent 5975C mass-selective detector operated in electron impact mode (EI, 70 eV), equipped with an Agilent 7693 autosampler, and an Agilent DB23 column (60 m length × 0.25 mm internal diameter × 0.15 μm film thickness) (Agilent Technologies, Santa Clara, CA, USA). Data analysis was carried out with the Agilent G1701EA MSD Productivity Chemstation software, revision E.02.00.

Derivatization of fatty acid methyl esters to dimethyl disulfide (DMDS) adducts was carried out according to the procedure described by Sansone et al. [37], as modified by Astudillo et al. [12]. Identification of 16:1 isomers was carried out by elution time of the different DMDS adducts (corresponding to the position of the double bond), and by specific fragments of the mass spectra generated by cleavage of the C-C bond that originally contained the double bond (ω-fragment, Δ-fragment and Δ-fragment with the loss of methanol). 

### 2.4. Liquid Chromatography/Mass Spectrometry (LC/MS) Analyses of Phospholipids

Lipids corresponding to 50 µg of cell homogenate protein were extracted according Bligh and Dyer [32]. The samples were re-dissolved in hexanes/2-propanol/water (42:56:2, *v*/*v*/*v*) and injected into a Thermo Fisher Scientific Dionex Ultimate 3000 high-performance liquid chromatograph equipped with an Ultimate HPG-3400SD standard binary pump and an Ultimate ACC-3000 autosampler column compartment (Thermo Fisher Scientific, Waltham, MA, USA). Separation was carried using a FORTIS HILIC column (150 mm × 3 mm, 3 µm particle size) (Fortis Technologies, Neston, UK). The mobile phase consisted of a gradient of solvent A (hexanes/isopropanol 30:40, *v*/*v*) and solvent B (hexanes/isopropanol/20 mM ammonium acetate in water, 30:40:7, *v*/*v*/*v*). The gradient started at 75% A from 0 to 5 min, then decreased from 75% A to 40% A at 15 min, from 40% A to 5% A at 20 min, was held at 5% until 40 min, and then increased to 75% at 41 min. Then, the column was re-equilibrated holding 75% A for an additional 14 min period before the next sample injection [38]. The flow rate through the column was fixed at 0.4 mL/min. The liquid chromatography system was coupled online to an Sciex QTRAP 4500 mass spectrometer equipped with a Turbo V Ion Source and a TurbolonSpray probe for electrospray ionization (AB Sciex, Framingham, MA, USA). Source parameters were set as follows: ion spray voltage, −4500 V; curtain gas, 30 psi; nebulizer gas, 50 psi; desolvation gas, 60 psi; temperature, 425 °C. Phospholipid species were analyzed in scheduled multiple reaction monitoring mode with negative ionization, detecting in Q3 the *m*/*z* of 253.2, corresponding to 16:1 fatty acids, as [M-H]^−^. The chromatographic peaks of each species were integrated and normalized to the peak area of the internal standard corresponding to each phospholipid class. 

### 2.5. FAHFA Analysis by LC/MS

FAHFA determination by LC/MS was carried out according to Zhang et al. [39], as modified by Rodríguez et al. [40]. Analyses were performed using a high-performance liquid chromatograph Thermo Fisher Scientific Dionex Ultimate 3000 equipped with an Ultimate HPG-3400SD standard binary pump and an Ultimate ACC-3000 Autosampler (Thermo Fisher Scientific, Waltham, MA, USA), coupled to a Sciex QTRAP 4500 Mass Spectrometer operated in negative ion mode (AB Sciex, Framingham, MA, USA). For the quantitative measurement of 16:1-derived FAHFA species, the instrument was set to multiple reaction monitoring mode, selecting three transitions for each FAHFA: parent ion → hydroxy fatty acid, parent ion → hydroxy fatty acid minus water loss and parent ion → *m*/*z* of esterified fatty acid as carboxylate ion. 

### 2.6. iPLA_2_-VIA Antisense Inhibition Studies

The iPLA_2_-VIA antisense oligonucleotide utilized in this work has been described in previous studies [41,42,43,44,45,46]. The iPLA_2_-VIA antisense sequence (5′-CTC CTT CAC CCG GAA TGG GT) corresponds to nucleotides 59–78 in the murine iPLA_2_-VIA sequence. As a control, the sense complement was used (5′-ACC CAT TCC GGG TGA AGG AG). Both sense and antisense oligonucleotides contained phosphorothioate linkages to limit degradation. The oligonucleotides were mixed with Lipofectamine RNAiMAX (Thermo Fisher Scientific, Walthman, MA, USA), following the manufacturer’s instructions. Oligonucleotide treatment and culture conditions were not toxic for the cells as assessed by the trypan blue dye exclusion assay and by quantitating adherent cellular protein. iPLA_2_ activity was measured exactly as described by Balboa et al. [29].

### 2.7. Data Analysis

The results are shown as means ± standard error of the mean and were analyzed for statistical significance by *t*-test (two groups) or by ANOVA (more than two groups), followed by Tukey’s post hoc test, using GraphPad Prism software (version 9.0.0, GraphPad Software, Inc., San Diego, CA, USA). A value of *p* < 0.05 was considered statistically significant. 

## 3. Results

### 3.1. Endogenous Distribution of 16:1 Fatty Acids among Macrophage Phospholipids

**Figure 1:**
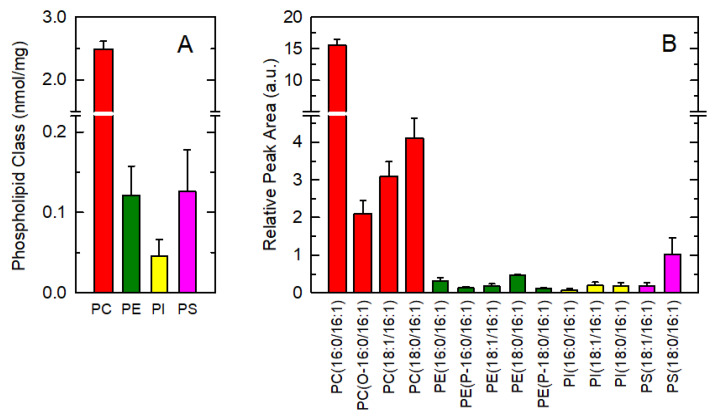
The 16:1-containing phospholipid species in resident peritoneal macrophages. (**A**) 16:1 content in phospholipids as shown by class. (**B**) Distribution profile of 16:1-containing choline-containing glycerophospholipids (PC) (red), ethanolamine-containing phospholipids (PE) (green), phosphatidylinositol (PI) (yellow), and phosphatidylserine (PS) (pink) species in macrophages, as determined by LC/MS. The results are shown as means ± standard error (*n* = 6).

Given the biological relevance of 16:1 fatty acids as endogenous molecules with anti-inflammatory properties, we began this work by assessing the cellular distribution of these fatty acids in murine resident peritoneal macrophages. Quantitative analysis of the distribution of 16:1 fatty acids within phospholipid classes was performed by gas chromatography coupled with mass spectrometry (GC/MS). Strikingly, more than 90% of total cellular 16:1 fatty acid was found in choline-containing glycerophospholipids (PC). Other major phospholipid classes such as ethanolamine-containing phospholipids (PE), phosphatidylinositol (PI) or phosphatidylserine (PS) contained only low amounts of this fatty acid (Figure 1A).

This phospholipid 16:1 fatty acid distribution is striking and prompted us to determine the distribution profile of 16:1-containing molecular species by using high-performance liquid chromatography coupled to a QTRAP 4500 triple quadrupole in multiple reaction mode (LC/MS). The 16:1-containing species were unequivocally identified by detecting the production of an *m*/*z* fragment of 253.2, corresponding to the [M-H]^−^ ion of 16:1. Following nomenclature recommendations, fatty chains within phospholipids are designated by their number of carbons:double bonds. A designation of O- before the first fatty chain indicates that the sn-1 position is ether-linked, whereas a P- designation indicates a plasmalogen form (sn-1 vinyl ether linkage) [47]. A total of 14 major species were detected, comprising ~90% of total cellular 16:1 (Figure 1B). Strikingly again, ~75% of total 16:1 in PC was found in a single phospholipid species, namely PC(16:0/16:1).

### 3.2. Decreased Levels of 16:1-Containing Phospholipid Species in Stimulated Macrophages

**Figure 2:**
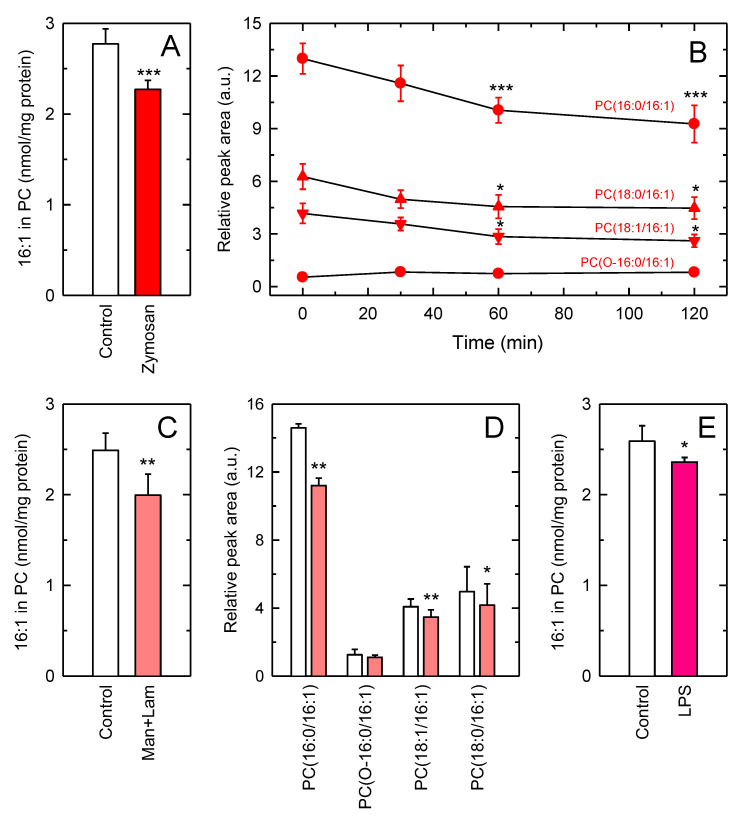
Effect of macrophage activation on the cellular levels of 16:1-containing PC. (**A**) The cells were either untreated (control) or stimulated with 1 mg/mL zymosan for 1 h. Afterward, the content of 16:1 fatty acid in PC was determined. These results are shown as means ± standard error (*n* = 6). *** *p* < 0.001, significance of zymosan-stimulated cells versus control cells. (**B**) Time-course of the effect of zymosan on the levels of 16:1-containing PC species. Results are shown as means ± standard error (*n* = 6). *** *p* < 0.001 or * *p* < 0.05, significance of zymosan-stimulated cells at the indicated times versus unstimulated conditions (zero time). (**C**) The cells were either untreated (control) or stimulated with 0.5 mg/mL mannan (Man) plus 0.5 mg/mL laminarin (Lam) for 1 h. Afterward, the content of 16:1 fatty acid in PC was determined. Results are shown as means ± standard error (*n* = 6). ** *p* < 0.01, significance of Man and Lam-stimulated cells versus control cells. (**D**) The different 16:1-containing PC species were measured in unstimulated cells (open bars) or cells stimulated with 0.5 mannan plus 0.5 mg/mL laminarin (red bars) for 1 h. Results are shown as means ± standard error (*n* = 6). ** *p* < 0.01 or * *p* < 0.05, significance of Man and Lam-stimulated cells versus control cells within each species. (**E**) Raw cells were either untreated (control) or stimulated with 200 ng/mL bacterial lipopolysaccharide (LPS) for 6 h. Afterward, the content of 16:1 fatty acid in PC was determined. Results are shown as means ± standard error (*n* = 6). * *p* < 0.05, significance of LPS-stimulated cells versus control cells.

The unexpected high amounts of 16:1 within one single species make it reasonable to speculate a role for this species in stimulus-response coupling. Consistent with this view, previous lipidomic work from our laboratory characterizing phospholipid changes in resting versus activated macrophages, identified PC (32:1) as a species whose levels decreased after activation [19]. This species was found to contain 16:1 at the sn-2 position, as assessed by fragmentation experiments using an ion-trap mass spectrometer [19]. To further extend these initial findings, we set out to measure the changes in the levels of all 16:1-containing PC species after activation. For these experiments, we used yeast-derived zymosan as a macrophage stimulus. Cellular stimulation significantly reduced the levels of all major 16:1-containing PC species in a time-dependent manner, with PC (16:0/16:1) experiencing the most pronounced decreases (Figure 2A,B). As for the rest of 16:1-containing phospholipid species not of the PC class, apart from being present at very low levels in comparison with PC species (Figure 1), no significant changes were appreciated upon zymosan stimulation. 

It is worth noting that zymosan preparations are yeast cell wall homogenates, and thus they may contain undefined amounts of 16:1 fatty acids. To prevent the lipid component of zymosan influencing the responses measured, we assayed a combination of the two major carbohydrate polymers present in zymosan, mannan (α-[1-6]-mannose) and laminarin (β-[1-3]-glucan), which are the responsible for activating phospholipid turnover pathways in macrophages via engagement of a variety of C-type lectin receptors [48]. The results, shown in Figure 2C,D, indicate that the combination of mannan and laminarin produced almost identical results to those obtained with zymosan, thus ruling out an effect of endogenous zymosan lipids on macrophage 16:1 fatty acid mobilization. Given that macrophages respond to a wide variety of stimuli, it was interesting to investigate next whether other stimuli of the innate immune response were able to also induce the hydrolysis of 16:1-containing PC phospholipids. Figure 2E shows show that stimulation of macrophages with bacterial lipopolysaccharide (LPS), a Toll-like receptor 4 agonist, did induce PC hydrolysis to an extent that was comparable to that found for zymosan or mannan plus laminarin. These data suggest that the reduction in 16:1-containing PC levels may constitute a hallmark of macrophage activation by innate stimuli.

### 3.3. Fatty Acid Isomers in the Response

**Figure 3:**
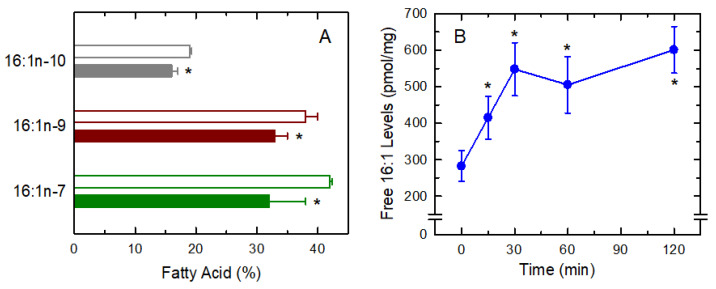
Release of 16:1 fatty acids in activated macrophages. (**A**) 16:1 fatty acid isomers were analyzed by GC/MS after transmethylation and transformation into dimethyl disulfide (DMDS) derivatives. The content of each isomer was analyzed in control unstimulated cells (open bars) or cells stimulated with mannan plus laminarin (0.5 mg/mL each) for 1 h. (**B**) Time-course of the release of 16:1 fatty acid from mannan plus laminarin-stimulated macrophages, as assessed by GC/MS after derivatizing the free fatty acids to methyl esters. Results are shown as means ± standard error (*n* = 6). * *p* < 0.05, significantly different from unstimulated cells.

In previous work from our laboratory, we showed that at least three 16:1 fatty acid positional isomers co-exist at significant levels in macrophages and other innate immune cells, namely 16:1n-7 (cis-9-hexadecenoic acid; palmitoleic acid), 16:1n-9 (cis-7-hexadecenoic acid; hypogeic acid), and 16:1n-10 (cis-6-hexadecenoic acid; sapienic acid), and that the three of them exhibit anti-inflammatory activity to different degrees [8,9]. It was therefore important to assess whether or not the three isomers are differentially released upon zymosan activation of the cells. Figure 3A shows that stimulated macrophages exhibited decreased amounts of all three isomers, suggesting that whatever may be the pathway for 16:1 mobilization, it does not distinguish the position of the double bond. In an analogous manner to arachidonic-acid containing phospholipids, the decreases of which after cellular stimulation are the result of increased free arachidonic acid release [49,50,51], we reasoned that the decrease in cellular 16:1-containing phospholipids is due to the release of 16:1 fatty acids during activation. As shown in Figure 3B, this was found to be the case, and a time-dependent increase in free 16:1 fatty acid formation could be detected after activation of the macrophages. At 1 h, 16:1 release had increased two-fold over unstimulated levels, which represents 10–15% of total fatty acid initially present in the cells. 

### 3.4. PLA_2_ Involvement in 16:1 Release

**Figure 4:**
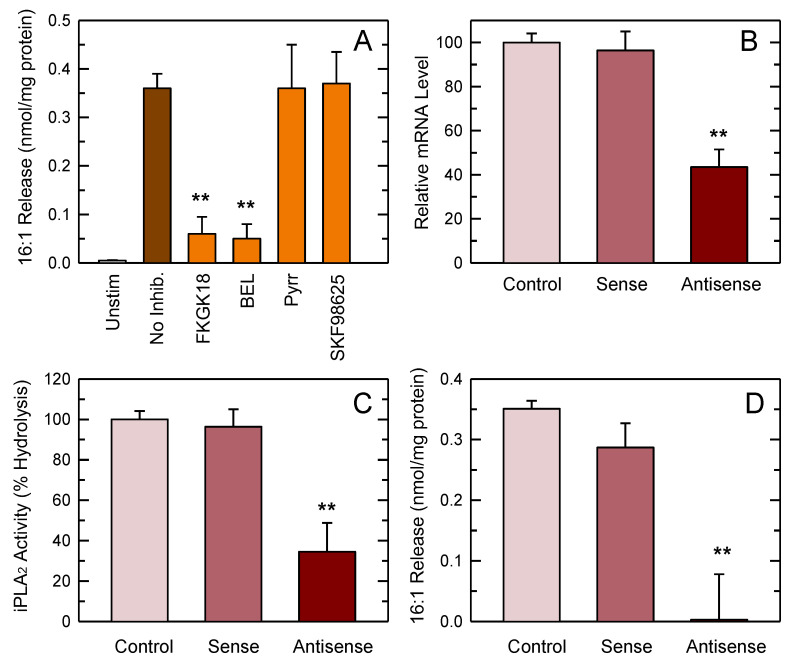
Release of 16:1 fatty acids by stimulated macrophages. (**A**) The cells were either unstimulated or stimulated by mannan plus laminarin (0.5 mg/mL each) for 1 h (colored bars) in the absence (no inhibition) or presence of the following inhibitors: 10 µM FKGK18, 5 µM bromoenol lactone (BEL), 2 µM pyrrophenone (pyrr), or 10 µM SKF98625. Afterward, total content of 16:1 fatty acids was measured by GC/MS. The fatty acid release was calculated by subtracting the amount of phospholipid-bound 16:1 in stimulated cells from that in unstimulated cells. Results are shown as mean values ± standard error of the mean. (*n* = 4). ** *p* < 0.01, significantly different from stimulated cells in the absence of inhibitors. (**B**) The cells were treated for 36 h with sense or antisense oligonucleotide, or vehicle (control), and calcium- (Ca^2+^)-independent group VIA phospholipase A_2_ (iPLA_2_-VIA) mRNA expression was analyzed by qPCR. (**C**) iPLA_2_ in homogenates from control, sense- or antisense-treated cells was determined by in vitro assay. (**D**) After the oligonucleotide treatments, the cells were stimulated and 16:1 release was determined. Results are shown as mean values ± standard error of the mean. (*n* = 4). ** *p* < 0.01, significantly different from control cells.

Since our previous lipidomic studies indicated that 16:1 fatty acids are primarily located at the sn-2 position of phospholipids in resident peritoneal macrophages [19], it would be logical to expect that a PLA_2_ mediates 16:1 fatty acid mobilization from the activated cells. In order to verify this point, we first tested the effect of selective inhibitors of the two major intracellular PLA_2_s potentially responsible for fatty acid release in activated macrophages, i.e., cPLA_2_α and iPLA_2_-VIA [19,20]. To inhibit cPLA_2_α, we used pyrrophenone. To inhibit iPLA_2_-VIA, we used the fluoroketone FKGK18 and the structurally unrelated compound bromoenol lactone (BEL). These inhibitors are the most potent and selective compounds currently available to block cPLA_2_α and iPLA_2_-VIA in cells [13,52]. In addition, we also tested the effect of SKF98625, an inhibitor of coenzyme A-independent transacylase, which acts in concert with cPLA_2_α to remodel phospholipids with polyunsaturated fatty acids [53]. Figure 4A shows that 16:1 fatty acid release was almost completely inhibited by the two iPLA_2_-VIA inhibitors, while pyrrophenone and SKF98625 had no effect. Under the conditions utilized, none of the inhibitors exerted any detectable effect on cell viability (Figure S1).

To confirm the results obtained with inhibitors, we next examined the effect of an iPLA_2_-VIA antisense oligonucleotide, which we and others have previously used with success to reduce the expression of this enzyme in a variety of cells [41,42,43,44,45,46]. Since we were unable to find reliable antibodies against murine iPLA_2_-VIA, the efficiency of antisense inhibition was analyzed by determining mRNA levels by qPCR, and also by assaying the BEL-inhibitable iPLA_2_ activity of cell homogenates after the different treatments. Using these techniques, we observed an mRNA decrease of 50–60% (Figure 4B), and an enzyme activity decrease of 60–70% (Figure 4C). Notably, even with this incomplete inhibition, the stimulus-induced mobilization of 16:1 fatty acids from the macrophages was almost completely abrogated (Figure 4D). Collectively, these data strongly suggest that iPLA_2_-VIA is the key enzyme regulating 16:1 fatty acid mobilization in activated macrophages. 

### 3.5. Studies with Deuterated Fatty Acids

An important question that arises from the results showing that activated macrophages release 16:1 is whether, after being released, the fatty acid stays in free fatty acid form or is utilized for the formation of new 16:1-containing lipids, which might also convey biological activity. It has been shown that some fatty acids released by activated macrophages, i.e., arachidonate and other polyunsaturates, are also transferred between various phospholipid species via remodeling reactions that are necessary to maintain the function and activity of the macrophages [53]. By analogy, we first set out to analyze whether the 16:1 released from PC is used for phospholipid fatty acid remodeling reactions, resulting in the enrichment of particular phospholipid species not of the PC class. To analyze the metabolic fate of each 16:1 isomer separately and also to improve detection by LC/MS, we utilized deuterated fatty acids for these experiments. 

**Figure 5:**
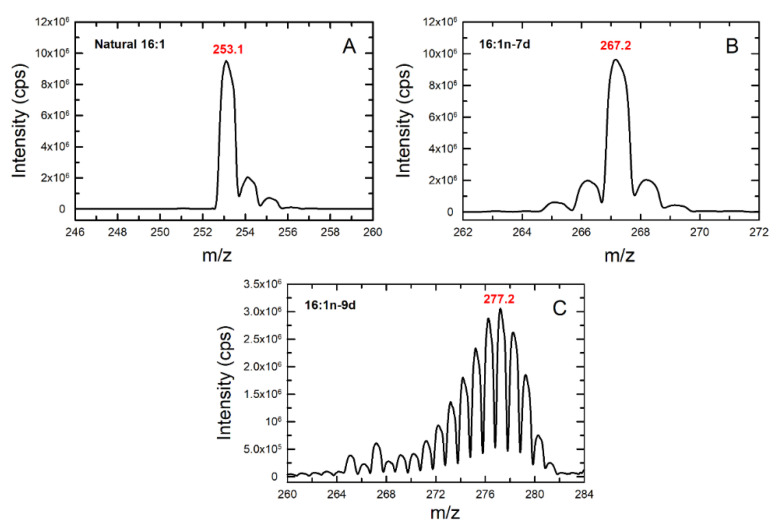
Detection of 16:1 isomers by mass spectrometry. Naturally occurring 16:1 (**A**), deuterated 16:1n-7 (**B**), and deuterated 16:1n-9 (**C**) were injected directly into the mass spectrometer and their *m*/*z* spectra were determined**.** The *m*/*z* of the most abundant ions are highlighted in red.

Figure 5 shows the characteristic profile of natural 16:1, deuterated 16:1n-7 (16:1n-7d) and deuterated 16:1n-9 (16:1n-9d). Deuterated 16:1n-10 was not available at the time of carrying out these experiments. 16:1n-7d was obtained from commercial sources and contained 14 deuterium atoms; thus, it produced a bell-shaped set of peaks due to the presence of various isotopomers with a maximum at *m*/*z* 267, corresponding to the *m*/*z* of the native molecule plus 14 deuteriums. 16:1n-9d was synthesized in our laboratory and contained 27 deuterium atoms (all hydrogen atoms bound to saturated carbons); thus, it produced a bell-shaped set of peaks with a maximum at *m*/*z* 277, that is, the *m*/*z* of native 16:1 plus 27. The signal produced by native 16:1 was very different, showing a decay from a maximum at *m*/*z* 253, allowing clear differentiation.

**Figure 6:**
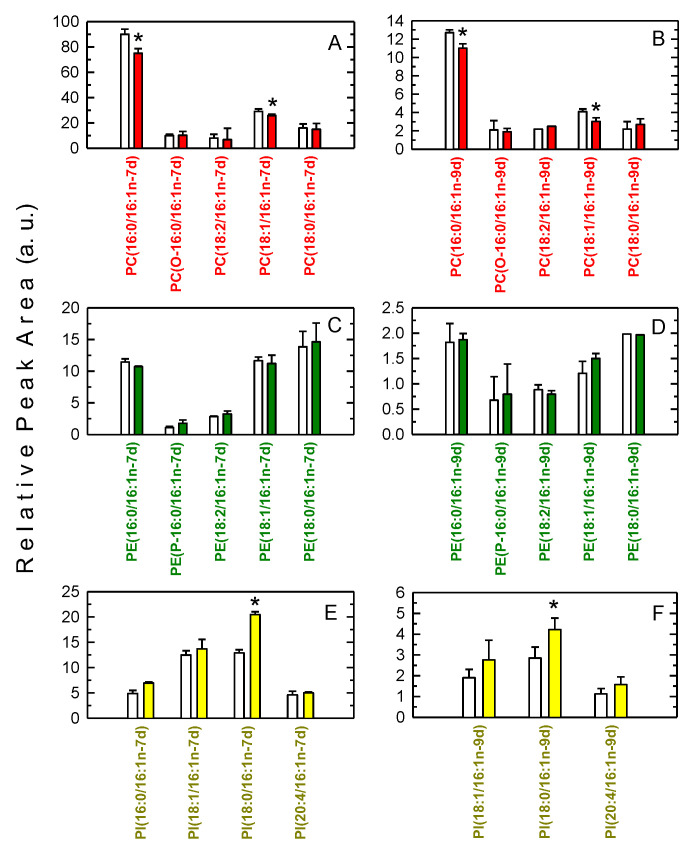
Analysis of phospholipid species containing deuterated 16:1 fatty acids. The cells were incubated with either deuterated 16:1n-7 (left column, panels **A**,**C**,**E**) or deuterated 16:1n-9 (right column, panels **B**,**D**,**F**) (10 µM, 1 h). After extensively washing the cells to remove the non-incorporated fatty acid, the cells were either untreated (open bars) or treated with mannan plus laminarin (0.5 mg/mL each, 1 h). The various deuterated species were determined by LC/MS. The results are shown as means ± standard error (*n* = 6). * *p* < 0.05, significantly different from untreated cells.

For experiments, the cells were spiked with either 16:1n-7d or 16:1n-9d for 1 h before stimulation for an additional 1 h. Exogenous deuterated fatty acids incorporated and distributed into phospholipids in a manner that closely resembled that of endogenous fatty acid (Figure 6).

For both 16:1n-7d and 16:1n-9d, it was observed that cellular stimulation promoted the loss of 16:1 from PC, with similar amounts of both fatty acids being lost. This is analogous to that previously found for endogenous 16:1 and thus confirms the validity of our metabolipidomic approach and, in turn, provides further evidence that stimulated 16:1 release is not isomer-specific. Importantly, a small but clearly significant increase in the levels of the species PI(18:0/16:1), containing either 16:1n-7 or 16:1n-9, could be detected (Figure 6). These results demonstrate that part of the 16:1 released from PC is transferred to PI during receptor-activation of the macrophages.

### 3.6. Synthesis of 16:1-Containing FAHFA

**Figure 7:**
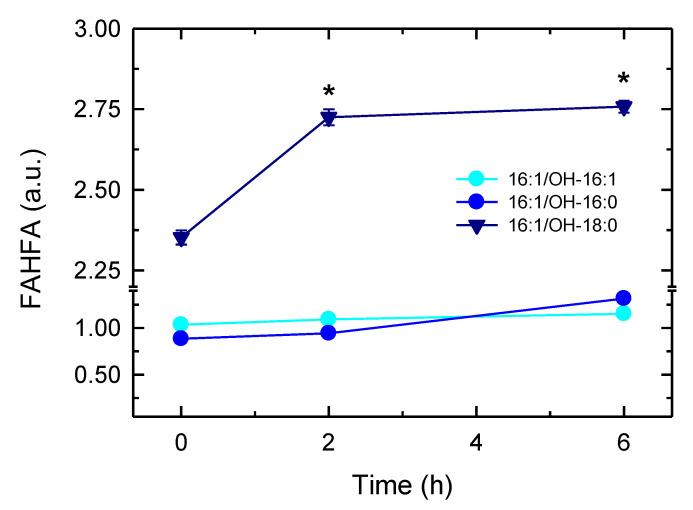
Identification of 16:1-derived branched fatty acid esters of hydroxy fatty acids (FAHFAs). The cells were stimulated with mannan plus laminarin (0.5 mg/mL each) for the indicated times. Then, 16:1-containing FAHFAs were determined by LC/MS. Three species were detected: hydroxyhexadecenoic acid (16:1/OH-16:1) (cyan), hydroxypalmitic acid (16:1/OH-16:0) (blue) and hydroxystearic acid (16:1/OH-18:0) (dark blue). A representative experiment is shown, and the data are expressed as mean values ± of three individual replicates. * *p* < 0.05, significantly different from unstimulated cells.

Recent work has described in mammalian cells the existence of a novel family of lipids consisting of a fatty acid esterified to the hydroxyl group of a hydroxy fatty acid (fatty acid esters of hydroxy fatty acids, FAHFA), with anti-diabetic and anti-inflammatory properties [21]. Since 16:1 has been shown to be one of the fatty acid constituents of FAHFA, we sought for the possible presence of 16:1-containing FAHFA in the activated macrophages. Three such species were detected in resting cells, namely the hexadecenoic acid esters of hydroxyhexadecenoic (16:1/OH-16:1), hydroxypalmitic, (16:1/OH-16:0) and hydroxystearic acids (16:1/OH-18:0) (Figure 7). Importantly, the latter clearly increased upon macrophage activation, indicating that part of the 16:1 released during the activation process is used to form compounds known to possess anti-inflammatory activity. 

## 4. Discussion

In the last few years, 16:1 fatty acids have been under the spotlight in bioactive lipid research as promising nutritional and metabolic biomarkers [54]. We have shown in this work that most of the 16:1 present in macrophages accumulates in just one phospholipid molecular species, namely PC(16:0/16:1), from which it is released under cell activation conditions. This is, to the best of our knowledge, the first study addressing the metabolism and dynamics of 16:1 fatty acids in cells in a comprehensive manner. While not focusing specifically on 16:1 fatty acids, recent studies that included lipidomic profiling of macrophages also identified the species PC(32:1), likely composed of 16:0 and 16:1, although other compositions are also possible (e.g., 14:0 and 18:1, or 18:0 and 14:1). Montenegro-Burke et al. [55] analyzed the lipid composition of polarized human monocyte-derived macrophages, finding six species that significantly differed among the various states—one of these was PC(32:1). Another study, also analyzing the glycerophospholipid profile of human macrophages, reported a decrease in monounsaturated phospholipids such as PC(32:1) during polarized activation of the cells to either M1 or M2 phenotypes [56]. In contrast, studies with murine macrophages reported increased levels of PC(32:1) following polarization with granulocyte/macrophage colony-stimulating factor and bacterial lipopolysaccharide [57,58].

The use of specific inhibitors together with antisense oligonucleotide approaches have allowed us to uncover the involvement of iPLA_2_-VIA in 16:1 mobilization. The other major intracellular PLA_2_ present in macrophages, cPLA_2_α, does not seem to participate in the process, as the specific inhibitor pyrrophenone had no discernible effect. The blockade of coenzyme A-independent transacylation also had no effect, indicating that 16:1 is not directly transferred between phospholipid species by mechanisms similar to those involving omega-6 fatty acids [53]. These results confirm and extend further our previous work on the role of iPLA_2_-VIA in mediating phospholipid fatty acid release responses in macrophages that do not involve arachidonic acid [19,20]. Moreover, the finding that the major substrate for iPLA_2_-VIA-mediated 16:1 release is PC(16:0/16:1) is also relevant in view of previous data showing that, in activated cells, iPLA_2_-VIA manifests selectivity for PC molecules containing palmitic acid at the sn-1 position, and that some of the major species hydrolyzed by the enzyme contain 16:1 at the sn-2 position [19,59]. Collectively, these results suggest that iPLA_2_-VIA is the key enzyme for 16:1 mobilization, and that the substrate selectivity of the enzyme in a whole cell context may be more stringent than what in vitro assays have suggested [60], probably due to cellular compartmentalization. Due to the anti-inflammatory character of the 16:1 fatty acids, it is conceivable that the regulated release of such fatty acids constitutes an important step for the mitigation or resolution of an inflammatory reaction. 

This study also addresses the involvement of part of the 16:1 liberated from PC in phospholipid fatty acid remodeling reactions. This is an important aspect in lipid signaling, as fatty acid remodeling reactions may increase phospholipid diversity and generate novel 16:1-containing phospholipid signatures that could be identified as fingerprints of specific activation states of the macrophages. More importantly, transfer of 16:1 moieties between phospholipids could lead to the generation of novel 16:1-containing species, or to increase the levels of pre-existing ones, which endow the cell with novel or improved functions. To explore this challenging possibility, we took advantage of the use of deuterated fatty acids. This strategy improves sensitivity and allows the characterization of the various isomers separately. Importantly, our approach identified a significant increase in the levels of a particular PI species, namely PI(18:0/16:1). This phenomenon was observed with the two deuterated fatty acids analyzed, and thus implies that the enzymes involved in the transfer do not distinguish positional isomers. Note, in this regard, that iPLA_2_-VIA also hydrolyzes all 16:1 isomers from PC equally well. 

It should be noted that the amount of 16:1 gained by the PI species is rather low, which suggests that 16:1 remodeling between phospholipids utilizes only small amounts of the fatty acid mobilized by iPLA_2_-VIA. Based on the relative peak areas of the samples and internal standards in LC/MS experiments, we estimate that as much as 5–10% of the 16:1 initially present in PC is transferred to PI. Notwithstanding, the fact that the cells actively make 16:1-containing PI strongly suggests that this lipid may have some signaling function. As a matter of fact, work by Koeberle et al. [61] has recently established that 16:1-containing PI possesses growth factor-like properties when added to fibroblast-like cell cultures. Clearly further work will be needed to explore a signaling role and possible cellular functions of 16:1-containing PI in macrophages. 

Our studies also unveil a second potentially important metabolic fate for the 16:1 mobilized from phospholipids in activated macrophages. The data show that part of the 16:1 hydrolyzed by iPLA_2_-VIA is diverted to the formation of FAHFA, a family of lipids with anti-inflammatory properties [21,62]. It is interesting that, of the three 16:1-containing FAHFA identified in resting macrophages, only one of them, 16:1/OH-18:0, decidedly increases in the activated cells. This suggests some sort of specificity in the formation of such species, which would be compatible with it playing a specific biological role. 

**Scheme 1:**
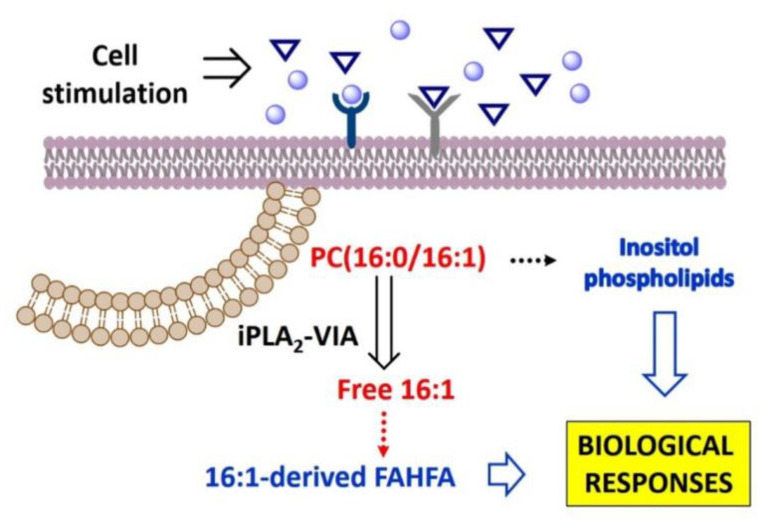
Dynamics of 16:1 fatty acid utilization by macrophages. Dotted arrows indicate the route may involve several undefined steps.

Similar to the situation discussed above regarding phospholipid 16:1 remodeling, formation of 16:1-containing FAHFA also appears to utilize small amounts of the fatty acid, accounting for as much 1% of total 16:1 mobilized from phospholipids. However, comparisons of the relative peak areas corresponding to the 16:1-containing FAHFA identified in our LC/MS experiments with that of a similar standard (palmitic acid ester of hydroxystearic acid) indicate that FAHFA levels increase in activated cells up to approximately 4 nM, a value that is in the same range as that reported for FAHFA levels in mice serum (0.4–2.5 nM), and also comparable to the range of signaling lipids such as eicosanoids and endocannabinoids [21]. Thus, it is possible that even at the levels identified in this study, 16:1-containing FAHFA may be of pathophysiological relevance (Scheme 1). Experimentation is currently underway to validate this intriguing possibility. 

## 5. Conclusions

Our study constitutes the first report addressing the dynamics of 16:1 utilization by macrophages. A key issue in this area of research is to determine whether the 16:1 fatty acid isomers exert their actions as free fatty acids per se or, instead, they are converted/incorporated into another lipid structure conveying the biological activity. We demonstrate that 16:1 fatty acids are released from phospholipids via an iPLA_2_-VIA-regulated pathway. Moreover, while most of the released fatty acids remain in free fatty acid form, small parts are utilized in phospholipid fatty acid remodeling reactions to enrich PI with 16:1, and also to form 16:1-containing FAHFA (Scheme 1). As both 16:1-containing PI and FAHFA are known to possess biological activity, our results would be compatible with the challenging possibility that (a) highly-regulated metabolite(s) of 16:1 mediate(s) at least part of the effects attributed to the fatty acid. Thus, our work provides new concepts and ideas that may help to explain the mechanisms through which the 16:1 fatty acids exert their anti-inflammatory actions. 

## Figures and Tables

**Scheme A1:**
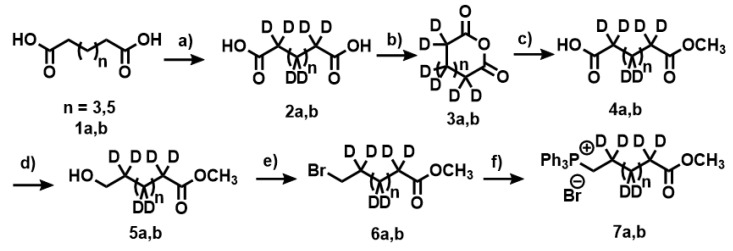
Synthesis of the first fragment for the Wittig reaction, deuterated heptanal as a phosphonium bromide salt (Wittig salt) (7a,b). Pimelic acid (1a,b) was used as starting material. Experimental conditions: (**a**) Pd(C) 10%, D_2_O, NaOD (40%), 260 °C, 30 bar; (**b**) AC_2_O, 150 °C; (**c**) CH_3_OH, 70 °C; (**d**) BH_3_THF; (**e**) CBr_4_ Ph_3_P; (**f**) Ph_3_P, CH_3_CN, 100 °C.

**Scheme A2:**
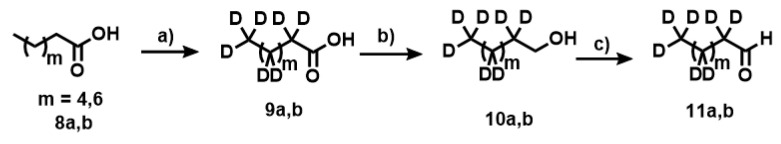
Synthesis of the second fragment for the Wittig reaction, deuterated nonanal (11a,b). Nonanoic acid (8a,b) was used as starting material. Experimental conditions: (**a**) Pd(C) 10%, D_2_O, NaOD (40%), 260 °C, 30 bar; (**b**) LiAlH_4_/THF; (**c**) PCC/DCM.

**Scheme A3:**
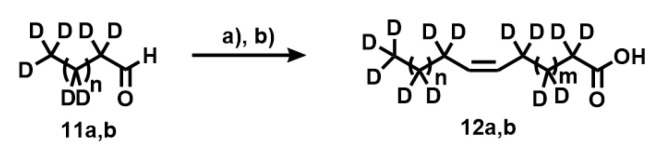
Synthesis of deuterated fatty acids. Coupling [D_17_]nonanal and [D_13_]heptanal with the phosphonium bromide achieved by heating in acetonitrile and cesium carbonate. The cis-specific Wittig reaction leads to the formation of the cis-double bond under mild conditions. Experimental conditions: (**a**) 7a,b, Cs_2_CO_3_, CH_3_CN; (**b**) LiOH, H_2_O, CH_3_OH.

## Supplementary Materials

The following are available online at https://www.mdpi.com/2227-9059/8/11/480/s1, Figure S1: PLA_2_ inhibitors do not affect macrophage viability.

## Abbreviations

GC/MS	Gas chromatography coupled to mass spectrometry
LC/MS	Liquid chromatography coupled to mass spectrometry
FAHFA	Branched fatty acid esters of hydroxy fatty acids
PC	Choline-containing glycerophospholipid
PE	Ethanolamine-containing glycerophospholipid
PI	Phosphatidylinositol
PS	Phosphatidylserine
PLA_2_	Phospholipase A_2_
cPLA_2_α	Cytosolic group IVA phospholipase A_2_
iPLA_2_-VIA	Calcium-independent group VIA phospholipase A_2_

## Appendix A

### Appendix A.1. Synthesis of Deuterated Fatty Acids

^1^H NMR (400 MHz) and ^13^C NMR (100.6 MHz) spectra were recorded in CDCl_3_ as solvent. Chemical shifts for protons are reported in ppm from TMS with the residual CHCl_3_ resonance as internal reference. Chemical shifts for carbons are reported in ppm from TMS and are reference to the carbon resonance of solvent. Data are reported as follows: chemical shift, multiplicity (s = singlet, d = doublet, t = triplet, q = quartet, m = multiplet, br = broad) coupling constants in Hertz, and integration. The high-resolution mass spectrometer used was Agilent 5973, ESI-QTOF. Flash chromatography was carried out using silica gel (230–240 mesh). Thin-layer chromatography analyses were performed using glass-backed plates coated with silica gel 60 and F254 indicator and visualized by either ultraviolet irradiation or by staining with phosphomolybdic acid solution.

The deuterated fatty acids were synthesized according to Darwish et al. [63] with modifications, as described below. The synthetic procedure uses two fragments that, through a Wittig reaction and final saponification, leads to deuterated fatty acids (Scheme A1, Scheme A2 and Scheme A3).

### Appendix A.2. Deuteration of Pimelic Acid (General Procedure)

A mixture of pimelic acid (7 g, 43.7 mmol), Pd(C) (10%) (1.1 g) and 40% *w*/*w* NaOD (8 g, 19.7 mmol) in D_2_O (70 mL) was loaded into the Parr pressure reactor. The contents of the reactor were degassed by purging with hydrogen gas and then sealed and heated to 260 °C (30 bar), with constant stirring for six days. The reactor was cooled to room temperature, and the contents were filtered off. The catalyst was washed with water (4 × 25 mL). The aqueous filtrate was acidified to pH 2 using 3 M HCl. The white solid that was formed upon acidification was extracted from the aqueous solution using ethyl acetate (5 × 100 mL) and washed with saturated NaCl. The organic layers were combined and dried with anhydrous MgSO_4_, and the solvent was removed under vacuum to give white solid (5.4 g, 72%). [D_10_]pimelic acid (94% D). ^1^H NMR (400 MHz, CDCl_3_): δ_H_ 1.36 (residual CH_2_), 1.60 (residual 2×CH_2_), 2.31 (residual 2×CH_2_). ^13^C NMR (100.6 MHz, CDCl3): δ_C_ 179.85 (2×CO_2_H). [D_14_]azelaic acid (92% D): ^1^H NMR (400 MHz, CDCl3): δ_H_ 1.30 (residual 3×CH2), 1.55 (residual 2×CH2), 2.28 (residual 2×CH_2_). ^13^C NMR (100.6 MHz, CDCl_3_): δ_C_ 178.80 (2×CO_2_H).

### Appendix A.3. Deuteration of Nonanoic Acid and Heptanoic Acid

[D_17_]nonanoic and [D_13_]heptanoic acids were prepared according to the general procedure. [D_17_]nonanoic acid (77%, 95% D): ^1^H NMR (400 MHz, CDCl3): δ_H_ 0.81 (residual CH3), 1.22 (residual 5×CH_2_), 1.57 (residual CH_2_), 2.29 (residual CH_2_). ^13^C NMR (100.6 MHz, CDCl_3_): δ_C_ 13.5 (CD_3_), 21.2 (CD_2_), 23.9 (CD_2_), 28.6 (3×CD_2_), 31.1 (CD_2_), 33.1 (CD_2_), 179.85 (CO_2_H). [D_13_]heptanoic acid (78%, 93% D): ^1^H NMR (400 MHz, CDCl3): δ_H_ 0.80 (residual CH_3_), 1.19 (residual 3×CH_2_), 1.59 (residual CH_2_), 2.31 (residual CH2). ^13^C NMR (100.6 MHz, CDCl3): δ_C_ 14.0 (CD_3_), 22.5 (CD_2_), 24.4 (CD_2_), 27.9 (CD_2_), 30.7 (CD_2_), 32.7 (CD_2_), 177.90 (CO_2_H).

### Appendix A.4. Synthesis of Anhydride 3a,b

A solution of [D_10_]pimelic acid 2a (8.16 g, 48 mmol) in acetic anhydride (170 mL) was refluxed for 4 h. The solution was cooled to room temperature and the solvent was removed at reduced pressure. Xylene (100 mL) was added, and the mix was stirred for 10 min at room temperature. The solvent was removed at reduced pressure to yield a white solid. (7.0 g, 97%): ^1^H NMR (400 MHz, CDCl_3_): δ_H_ 1.40 (residual CH_2_), 1.65 (residual 2×CH_2_), 2.42 (residual 2×CH_2_). ^13^C NMR (100.6 MHz, CDCl_3_): δ_C_ 19.9 (CD_2_), 22.3 (2×CD_2_), 36.4 (2×CD_2_), 168.68 (CO_2_^−^). [D_14_]azelaic anhydride (95%): ^1^H NMR (400 MHz, CDCl_3_): δ_H_ 1.37 (residual 3×CH_2_), 1.61 (residual 2×CH_2_), 2.38 (residual 2×CH_2_). ^13^C NMR (100.6 MHz, CDCl3): δ_C_ 19.9 (CD_2_), 21.2 (2×CD_2_), 27.5 (2×CD_2_), 32.7 (2×CD_2_), 167.53 (2×CO_2_^−^).

### Appendix A.5. Synthesis of Compounds 4a,b

A solution of [D_10_]pimelic anhydride 3a (11.25 g, 74.0 mmol) in anhydrous methanol (110 mL) was refluxed under argon for 3 h. The solvent was removed in vacuo. The resulting liquid (13.29 g, 72.22 mmol, 98%) was used in the next step. ^1^H NMR (400 MHz, CDCl_3_): δ_H_ 1.27 (residual CH_2_), 1.54 (residual 2×CH_2_), 2.29 (residual 2×CH_2_), 3.58 (s, 3H, CH_3_O^−^). ^13^C NMR (100.6 MHz, CDCl_3_): δ_C_ 24.1 (CD_2_), 28.1 (2×CD_2_), 34.4 (2×CD_2_), 51.29 (CH_3_O^−^), 174.21 (CO_2_^−^), 179.54 (CO_2_H). Compound 4b (97%): ^1^H NMR (400 MHz, CDCl3): δ_H_ 1.24 (residual 3×CH2), 1.60 (residual 2×CH_2_), 2.29 (residual 2×CH_2_), 3.56 (s, 3H, CH_3_O^−^) ^13^C NMR (100.6 MHz, CDCl_3_): 20.1 (CD_2_), 22.8 (2×CD_2_), 28.1 (2×CD_2_), 32.3 (2×CD_2_), δ_C_ 51.09 (CH_3_O^−^), 173.71 (CO_2_^−^), 179.74 (CO_2_H).

### Appendix A.6. Synthesis of Compounds 5a,b

Borane-methyl complex (41 mL, 81.9 mmol) was added dropwise to a stirred solution of 4a (12.15 g, 66.03 mmol) in anhydrous THF (82 mL) at 0 °C under an inert atmosphere. The solution was maintained at 0 °C for 2 h and then allowed to warm up gradually to room temperature overnight. MeOH (10 mL) was added dropwise and the mixture was stirred for 2 h. The solvent was removed in vacuo and water and ethyl acetate were added to the residue, and the aqueous layer was extracted twice more with ethyl acetate. The combined organic layer was washed with NaHCO_3_, brine and H_2_O, dried over MgSO_4_ and the solvent removed in vacuo to yield a clear liquid. (7.8 g, 70%). ^1^H NMR (400 MHz, CDCl3): δ_H_ 1.24 (residual 2×CH_2_), 1.50 (residual CH_2_), 2.18 (residual CH_2_), 2.39 (residual CH_2_), 3.48 (br s, 2H, CH_2_OH), 3.56 (s, 3H, CH_3_O^−^). ^13^C NMR (100.6 MHz, CDCl_3_): δ_C_ 23.3 (3×CD_2_), 29.1 (CD_2_), 31.9 (CD_2_), 51.29 (CH_3_O^−^), 62.33 (CH_2_OH), 174.21 (CO_2_^−^). Compound 5a: ^1^H NMR (400 MHz, CDCl_3_): δ_H_ 1.22 (residual 4×CH_2_), 1.53 (residual 2×CH_2_), 2.13 (residual CH_2_), 3.45 (s, 2H, CH_2_OH), 3.58 (s, 3H, CH_3_O^−^). ^13^C NMR (100.6 MHz, CDCl_3_): δ_C_ 19.8 (4×CD_2_), 27.4 (CD_2_), 34.2 (CD_2_), 51.09 (CH_3_O^−^), 62.54 (CH_2_OH), 174.45 (CO_2_^−^).

### Appendix A.7. Synthesis of Compounds 6a,b

Triphenylphosphine (13.6 g, 51.8 mmol) was added to a solution of ethyl [D_10_]7-hydroxyheptanoate (7.8 g, 46.2 mmol) and CBr_4_ (16.6 g, 50.0 mmol) in dichloromethane (50 mL) at 0 °C under an inert atmosphere. The mixture was stirred overnight. The solvent was removed in vacuo and the residue was triturated with heptane (70 mL). The solid was removed by filtration and the solvent was passed through a Celite pad. The solvent was removed in vacuo to yield a clear liquid (9.75 g, 91%). ^1^H NMR (400 MHz, CDCl_3_): δ_H_ 1.29 (residual CH_2_), 1.42 (residual CH_2_), 1.59 (residual CH_2_), 1.81 (residual CH_2_), 2.26 (residual CH_2_), 3.37 (br s, 2H, CH_2_), 3.65 (s, 3H, CH_3_O^−^). ^13^C NMR (100.6 MHz, CDCl_3_): δ_C_ 22.3 (CD_2_), 27.4 (CD_2_), 33.58 (CH_2_Br), 51.30 (CH_3_O^−^), 174.21 (CO_2_^−^). Compound 6b (90%): ^1^H NMR (400 MHz, CDCl_3_): δ_H_ 1.34 (residual 3×CH_2_), 1.40 (residual CH_2_), 1.53 (residual CH_2_), 1.83 (residual CH_2_), 2.28 (residual CH_2_), 3.35 (s, 2H, CH_2_), 3.61 (s, 3H, CH_3_O^−^) 13C NMR (100.6 MHz, CDCl3): δ_C_ 18.3 (2×CD_2_), 22.0 (2×CD_2_), 28.1 (2×CD_2_), 33.48 (CH_2_Br), 51.38 (CH_3_O^−^), 174.44 (CO_2_^−^).

### Appendix A.8. Synthesis of Compounds 7a,b (Wittig Salt)

Anhydrous triphenylphosphine (27.4 g, 104.5 mmol) was added to a solution of ethyl [D_10_]7-bromoheptanoate (9.7 g, 41.8 mmol) and K_2_CO_3_ (1 g, 7.2 mmol) in acetonitrile (170 mL) at 0 °C under an inert atmosphere. The mixture was refluxed overnight. The solvent was removed in vacuo and the residue was triturated with methanol (60 mL). The Wittig salt was purified by flash chromatography (silica gel, eluent: DCM/methanol 20:1) to yield a clear oil. (7.0 g, 34%). ^1^H NMR (400 MHz, CDCl_3_): δ_H_ 1.25 (residual CH_2_), 1.48 (residual CH_2_), 1.54 (residual CH_2_), 1.77 (residual CH_2_), 2.22 (residual CH_2_), 3.60 (s, 3H, CH_3_O^−^), 3.74 (m, 2H, CH_2_P), 7.59–7.92 (m, 15H). ^13^C NMR (100.6 MHz, CDCl_3_): δC 22.3 (CD_2_), 22.53 (CH_2_P), 25.9 (CD_2_), 28.8 (CD_2_), 25.1 (CD_2_), 33.7 (CD_2_), 51.68 (CH_3_O^−^), 118.38 (Ar), 119.2 (Ar), 130.42 (Ar), 131.3 (Ar), 133.65 (Ar), 134.92 (Ar), 137.5 (Ar), 174.21 (CO_2_^−^). Compound 7b (41%): ^1^H NMR (400 MHz, CDCl_3_): δ_H_ 1.28 (residual 2×CH_2_), 1.47 (residual 2×CH_2_), 1.53 (residual CH_2_), 1.79 (residual CH_2_), 2.24 (residual CH_2_), 3.64 (s, 3H, CH_3_O^−^), 3.72 (m, 2H, CH_2_P), 7.53–7.5 (m, 15H). ^13^C NMR (100.6 MHz, CDCl_3_): δ_C_ 21.9 (CD_2_), 22.43 (CH_2_P), 25.4 (CD_2_), 28.6 (3×CD_2_), 25.5 (CD_2_), 33.9 (CD_2_), 51.88 (CH_3_O^−^), 118.58 (Ar), 119.3 (Ar), 130.7 (Ar), 131.2 (Ar), 133.65 (Ar), 134.82 (Ar), 138.3 (Ar), 174.31 (CO_2_^−^).

### Appendix A.9. Synthesis of Compounds 10a,b

A solution of 7a (28.5 g, 162.4 mmol) in THF (260 mL) was added dropwise at 0 °C to a suspension of LiAlH_4_ (12.3 g, 324.8 mmol) in anhydrous THF (350 mL). The mixture was refluxed for 3 h with vigorous stirring. The reaction was quenched by addition of NaOH (12.3 mL, 2 M), water (12.3 mL) and NaOH (36.9 mL, 2 M). The white solid was filtrated off. The solvent was removed in vacuo to yield a clear liquid. (23.8 g, 90%). ^1^H NMR (400 MHz, CDCl_3_): δ_H_ 0.82 (residual CH_3_), 1.22 (residual 6×CH_2_), 1.48 (residual CH_2_), 3.58 (s, 2H, CH_2_OH). ^13^C NMR (100.6 MHz, CDCl_3_): δ_C_ 13.8 (CD_3_), 22.1 (CD_2_), 25.2 (CD_2_), 29.3 (3×CD_2_), 30.1 (CD_2_), 32.2 (CD_2_), 62.73 (CH_2_OH). Compound 10b (88%): ^1^H NMR (400 MHz, CDCl_3_): δ_H_ 0.80 (residual CH_3_), 1.25 (residual 7×CH_2_), 1.48 (residual 2×CH_2_), 3.55 (s, 2H, CH_2_OH). ^13^C NMR (100.6 MHz, CDCl_3_): δ_C_ 14.0 (CD_3_), 22.5 (CD_2_), 25.7 (CD_2_), 29.6 (CD_2_), 31.7 (CD_2_), 33.6 (CD_2_), 62.53 (CH_2_OH).

### Appendix A.10. Synthesis of Compounds 11a,b

A solution of [D_17_]1-nonanol (5 g, 31 mmol) was added to a suspension of pyridinium chlorochromate (1.0 g, 46.6 mmol) and silica gel (10 g) in DCM (62 mL). Once the reaction was completed, diethyl ether (200 mL) was added and filtered over silica gel. The solvent was removed in vacuo to yield a yellow liquid. The aldehyde was distilled under reduced pressure to obtain a clear liquid. (110 °C, 15.5 torr, 2.4 g, 48%). ^1^H NMR (400 MHz, CDCl_3_): δ_H_ 0.80 (residual CH_3_), 1.22 (residual 5×CH_2_), 1.56 (residual CH_2_), 2.35 (residual CH_2_), 9.69 (s, 1H, CHO). ^13^C NMR (100.6 MHz, CDCl_3_): δ_C_ 14.0 (CD_3_), 22.7 (2×CD_2_), 29.4 (4×CD_2_), 43.7 (CD_2_), 202.73 (CHO). Compound 11b (50%): ^1^H NMR (400 MHz, CDCl_3_): δ_H_ 0.83 (residual CH_3_), 1.25 (residual 3×CH_2_), 1.53 (residual CH_2_), 2.55 (residual CH_2_), 9.55 (s, 1H, CHO). ^13^C NMR (100.6 MHz, CDCl_3_): δ_C_ 13.9 (CD_3_), 22.5 (CD_2_), 28.4 (2×CD_2_), 31.8 (CD_2_), 43.5 (CD_2_), 0.53 (CHO).

### Appendix A.11. Synthesis of Compounds 12a,b

[D_17_]nonanal (0.27 g, 1.7 mmol) was added at room temperature to a mixture of phosphonium salt 7a (0.84 g, 1.7 mmol) in dichloromethane (30 mL) and Cs_2_CO_3_ (2.25 g, 7 mmol) under inert atmosphere. The mixture was refluxed for 24 h with vigorous stirring. The progress of the reaction was monitored by thin-layer chromatography on silica gel using ethyl acetate-heptane 1:10 (Rf = 0.25). When the reaction was completed, it was cooled at room temperature, and the mixture was filtered through a Celite pad, which was then washed with DCM. The solvent was evaporated under reduced pressure and purified on a column of silica gel to obtain a methyl ester as a clear liquid. (0.4 g, 79%). ^1^H NMR (400 MHz, CDCl_3_): δ_H_ 0.73 (residual CH_3_), 1.21 (residual 8×CH_2_), 1.55 (residual CH_2_), 1.90 (residual CH_2_), 2.25 (residual CH_2_), 3.66 (s, 3H, CH_3_O^−^), 5.33 (m, 2H, HC=C). ^13^C NMR (100.6 MHz, CDCl_3_): δ_C_ 14.0 (CD_3_), 22.7 (CD_2_), 25.3 (CD_2_), 27.8 (3×CD_2_), 29.6 (4×CD_2_), 31.8 (CD_2_), 33.7 (CD_2_), 51.30 (CH_3_O^−^), 129.1 (HC=C), 129.9 (HC=), 174.60 (CO_2_^−^). Methyl [D_27_](Z)-9-hexadecenoic ester (69%). ^1^H NMR (400 MHz, CDCl_3_): δ_H_ 0.75 (residual CH_3_), 1.25 (residual 8×CH_2_), 1.57 (residual CH_2_), 1.89 (residual CH_2_), 2.27 (residual CH_2_), 3.63 (s, 3H, CH_3_O^−^). ^13^C NMR (100.6 MHz, CDCl_3_): δ_C_ 13.8 (CD_3_), 22.8 (CD_2_), 25.0 (CD_2_), 27.7 (2×CD_2_), 29.5 (5×CD_2_), 31.9 (CD_2_), 33.8 (CD_2_), 51.22 (CH_3_O^−^), 129.2 (HC=C), 130.0 (HC=), 174.50 (CO_2_^−^).

A solution of lithium hydroxide (90 mL, 1.6 M) was added to the methyl ester (2.5 g, 8.8 mmol) in methanol (80 mL) with stirring. The hydrolysis reaction was monitored by thin-layer chromatography. The reaction reached completion after 24 h. The aqueous layer was washed with diethyl ether (3 × 75 mL) and HCl (1 M) was added to the reaction mixture to acidify it to pH 1. The turbid aqueous solution was extracted three times with diethyl ether. The organic phase was washed once with brine and dried over magnesium sulfate. The solvent was evaporated under reduced pressure to give a pale-yellow oil. The oil was purified on a silica gel column (eluent heptane:ethyl acetate) to give a colorless oil of deuterated acid. (2.1 g, 8.3 mmol, 94%). ^1^H NMR (400 MHz, CDCl_3_): δ_H_ 0.83 (residual CH_3_), 1.30 (residual 8×CH_2_), 1.65 (residual CH_2_), 1.92 (residual CH_2_), 2.31 (residual CH_2_), 5.30 (m, 2H, HC=C). ^13^C NMR (100.6 MHz, CDCl_3_): δ_C_ 14.0 (CD_3_), 22.5 (CD_2_), 25.1 (CD_2_), 27.6 (3×CD_2_), 29.3 (4×CD_2_), 31.5 (CD_2_), 33.8 (CD_2_), 51.32 (CH_3_O^−^), 129.0 (HC=C), 129.8 (HC=), 174.63 (CO_2_^−^). Compound 12b (69%). ^1^H NMR (400 MHz, CDCl_3_): δ_H_ 0.77 (residual CH_3_), 1.27 (residual 8×CH_2_), 1.55 (residual CH_2_), 1.87 (residual CH_2_), 2.29 (residual CH_2_), 3.60 (s, 3H, CH_3_O^−^). ^13^C NMR (100.6 MHz, CDCl_3_): δ_C_ 13.9 (CD_3_), 22.6 (CD_2_), 25.1 (CD_2_), 27.9 (2×CD_2_), 29.1 (5×CD_2_), 31.4 (CD_2_), 33.5 (CD_2_), 51.22 (CH_3_O^−^), 129.3 (HC=C), 130.0 (HC=), 174.55 (CO_2_^−^).
