# Supplementary material for: Release of Anti-Inflammatory Palmitoleic Acid and Its Positional Isomers by Mouse Peritoneal Macrophages
Source: Biomedicines. 2020 Nov 6;8(11):480. doi: 10.3390/biomedicines8110480 (PMC7694668; doi:10.3390/biomedicines8110480)
Supplement: Supplementary file 1 [file biomedicines-08-00480-s001.pdf]

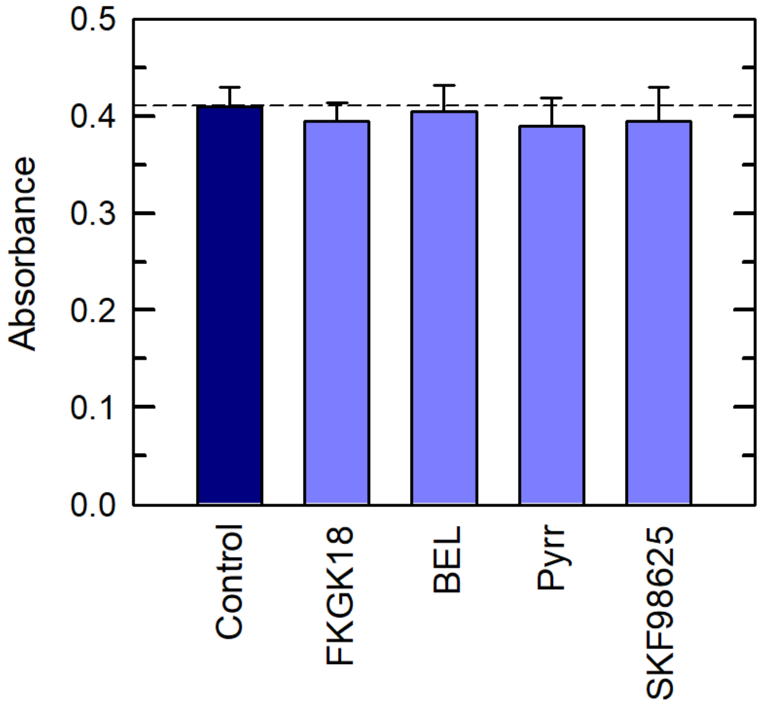

Figure S1. PLA_2 inhibitors do not affect macrophage viability. The cells were either untreated (Control) or treated with the following inhibitors for 1 h: 10 μM FKGK18, 5 μM bromoenol lactone (BEL), 2 μM pyrrophenone (pyrr), or 10 μM SKF98625. Afterward, cell viability was determined using the CellTiter 96® AQueous One Solution Cell Proliferation Assay kit (Promega, Madison, WI, USA), following the manufacturer's instructions, and absorbance was read at 490 nm. Results are shown as means ± S.E.M. (n=6).
